# Fish oil supplements in New Zealand are highly oxidised and do not meet label content of n-3 PUFA

**DOI:** 10.1038/srep07928

**Published:** 2015-01-21

**Authors:** Benjamin B. Albert, José G. B. Derraik, David Cameron-Smith, Paul L. Hofman, Sergey Tumanov, Silas G. Villas-Boas, Manohar L. Garg, Wayne S. Cutfield

**Affiliations:** 1 Liggins Institute, University of Auckland, Auckland, New Zealand; 2 Centre for Microbial Innovation, School of Biological Sciences, University of Auckland, Auckland, New Zealand; 3 Nutraceuticals Research Group, University of Newcastle, Callaghan, New South Wales, Australia

## Abstract

We evaluated the quality and content of fish oil supplements in New Zealand. All encapsulated fish oil supplements marketed in New Zealand were eligible for inclusion. Fatty acid content was measured by gas chromatography. Peroxide values (PV) and anisidine values (AV) were measured, and total oxidation values (Totox) calculated. Only 3 of 32 fish oil supplements contained quantities of eicosapentaenoic acid (EPA) and docosahexaenoic acid (DHA) that were equal or higher than labelled content, with most products tested (69%) containing <67%. The vast majority of supplements exceeded recommended levels of oxidation markers. 83% products exceeded the recommended PV levels, 25% exceeded AV thresholds, and 50% exceeded recommended Totox levels. Only 8% met the international recommendations, not exceeding any of these indices. Almost all fish oil supplements available in the New Zealand market contain concentrations of EPA and DHA considerably lower than claimed by labels. Importantly, the majority of supplements tested exceeded the recommended indices of oxidative markers. Surprisingly, best-before date, cost, country of origin, and exclusivity were all poor markers of supplement quality.

Fish oils are among the most popular dietary supplements in the world. In the United States, more than a third of the 17.7% of adults who use dietary supplements take fish oil^1^,^2^. Fish oils contain significant quantities of omega-3 long-chain polyunsaturated fatty acids (n-3 PUFA), including eicosapentaenoic acid (EPA) and docosahexaenoic acid (DHA) that are considered to be the metabolically active compounds^3^. Consumers take fish oil supplements for a variety of reasons, but particularly because it has shown promising effects of lowering inflammation^4^, improving cognition^5^, and lowering cardiovascular disease risk^6^.

n-3 PUFA are highly prone to oxidation due to the large number of double bonds within the fatty acid chain^7^,^8^. As fish oils oxidise, unoxidised fatty acids diminish and are replaced by a complex ‘soup' of lipid peroxides and secondary oxidation products (aldehydes and ketones)^7^. Addition of antioxidants to fish oils reduces but does not prevent oxidation^9^.

While specific oxidation species are difficult to measure, the degree of oxidation can be described by measuring the peroxide value (PV) and the anisidine value (AV). PV reflects the primary oxidation products (lipid peroxides) while AV reflects secondary oxidation products (aldehydes and ketones). Together, these parameters are used to estimate the total oxidation value (Totox). A number of organisations have recommended maximum levels for these indices^10^,^11^,^12^. However, these industry standards are based on palatability, as there are insufficient data to set standards based on health effects^13^.

When over-the-counter fish oil supplements have been investigated, the reported frequency of excessive oxidation has been highly variable^14^. A 2007 survey of retail fish oil supplements in New Zealand showed that 4 of 29 products tested had a Totox value greater than recommended levels, but limited information was provided about specific oxidative markers or study methodology^15^. In addition, 5 of the 29 products contained less n-3 PUFA than labelled^15^. Thus, in order to fill an existing knowledge gap, we aimed to comprehensively evaluate the fish oil supplements currently available in the New Zealand market, measuring simple oxidative markers as well as concentrations of n-3 PUFA.

## Methods

All encapsulated fish oil supplements sold at retail or online stores in New Zealand were eligible for inclusion in the study. Marine oils from other sources (such as krill, calamari, or algae, which have important differences in composition) fell outside the scope of this study and were not included. We selected encapsulated oils because this is the most common medium for consumption of fish oil supplements. If there were multiple fish oil supplements available from a particular company, all were included in the study, provided there was a claimed difference in oil composition. One vial of each supplement was purchased for sampling. Where products were found in multiple stores, the first identified vial with a best before date between 12 and 24 months from the time of purchase was selected, otherwise vials 9 to 30 months prior to best before date were eligible.

The recommended retail price and best before date were recorded. Immediately after purchase, oil capsules were removed from their packaging, stored in amber glass jars that were numbered and sealed, and placed inside a cardboard box. The box was then stored at 4°C. Thus, the oil capsules were stored in conditions expected to minimise oxidation^7^. All products were subsequently tested for the oil content, n-3 PUFA composition, PV, and AV. All tests and data analyses were performed by investigators who were blinded to the identity of individual supplements.

### Mass of oil content

Capsule oil mass was measured by weighing the oil capsule (m_1_), piercing and evacuating the oil contents, then washing the halved capsule in hexane. After drying the capsule shell, it was re-weighed (m_2_). Mass of capsule content was calculated as: m_1_ – m_2_. This procedure was performed in triplicate.

A 12 ml sample of oil was produced by combining the contents of 8–24 capsules (depending on capsule size). From this pooled oil sample, PV, AV, Totox, and fatty acid concentration were measured in triplicate.

### PV, AV, and Totox

PV was measured according to the European pharmacopoeia^16^ by visual titration of iodine. 2.5 g of oil was weighed in a volumetric flask. 50 ml of 3:2 (glacial acetic acid:trimethylpentane) was added with 500 μl of saturated potassium iodide solution. The flask was occluded and vigorously shaken for 60 seconds, and 30 ml of water added. The yellowed solution was titrated with 0.01 N sodium thiosulphate solution until almost colourless, when 500 μl of 1% starch solution was added. The resulting solution was titrated until colourless using 0.01 N sodium thiosulphate solution. The sodium thiosulphate volume was used to calculate the peroxide value in meq/l according to the formula “PV = [10 × (V − V_control_)]/m”, where *m* is the oil mass previously obtained. Based on triplicate measures, the intra- and inter-assay coefficients of variation were 2.6 and 1.8%, respectively.

AV was measured according to the European pharmacopoeia^16^ using absorbance spectrophotometry after reaction with p-anisidine. Briefly, 0.2 g of oil was weighed into a small vial. 10 ml of trimethylpentane was added. The A_1_ absorbance was measured at 350 nm against a reference solution of trimethylpentane. 1 ml of 2.5 g/l p-ansidine in acetic acid was added to 5 ml of the oil-trimethylpentane solution. After 10 minutes, the A_2_ absorbance was measured against a reference solution of 5 ml trimethylpentane with 1 ml of p-anisidine in acetic acid. AV was calculated from these absorbances and the oil mass (m) previously obtained: “10 × [1.2 × (A_2_ − A_1_)]/m”. Based on triplicate measures, the intra- and inter-assay coefficients of variation were 3.0 and 3.8%, respectively. Totox was subsequently calculated by the formula “(2 × PV) + AV”^12^.

Markers of oxidation were compared to international guidelines, with recommended values for PV, AV, and Totox of <5, <20, and <26 meq/l, respectively^10^,^11^,^12^.

### n-3 PUFA composition

1 ml of fish oil was frozen at −80°C for storage prior to analysis. 50 mg of oil samples were weighed out into solvent washed methylation tubes and dissolved in 10 ml of toluene. From this, 200 ul (1.0 mg) was taken for trans-methylation followed by gas chromatographic analysis. Briefly, 2 ml of methanol:toluene (4:1 v/v, containing C19:0, 20 μg/ml as internal standard) was added to the sample. Acetyl chloride (200 μl) was added while vortexing and the contents were heated for 1 hour at 100°C. The tubes were cooled in water (5 minutes) and K_2_CO_3_ 6% (5 ml) added and centrifuged (3000 × g, 5 min, 4°C). The upper toluene phase was collected and stored in a gas chromatograph (GC) vial at −20°C for analysis.

Methylated fatty acid samples were analysed by gas chromatography using a fixed carbon-silica column 30 m × 0.25 mm (DB-225) (J&W Scientific, Folsom, CA, USA). The GC was equipped with a flame ionization detector, auto-sampler and auto-detector. Injector and detector ports were set at 250°C. Oven temperature was programmed: 170°C for two minutes, increased 10°C/minute up to 190°C where it remained stationary for one minute. Temperature then increased 3°C/minute up to 220°C, which was maintained for a total run time of 30 minutes per sample. A split ratio of 10:1 and an injection volume of 3 μl was used. A known fatty acid mixture was used to identify peaks according to retention time and their concentration was determined using a 6890 Series gas chromatograph (Hewlett Packard, Palo Alto, CA, USA) with Chemstations Version A. 04.02 (Chemstations Inc, Houston, TX, USA)^17^. Each oil sample was analysed in triplicate.

The mass of individual fatty acids in each capsule were calculated in mg/capsule by the formula “specific fatty acid content = specific fatty acid concentration × capsule oil mass”.

### Data analyses

Adjusted values for PV, AV, and Totox were also calculated to account for the higher concentration of n-3 PUFA in some oils. Oils with a higher concentration of n-3 PUFA have more substrate for oxidation, and are therefore expected to have higher levels of oxidation markers. Adjusted values standardised the oxidative indices, reflecting the exposure to oxidised compounds per 300 mg of combined EPA + DHA (the most frequent labelled dose per capsule). This adjustment may provide a better estimate of a consumer's exposure to oxidation products, as less capsules of a more concentrated fish oil would be ingested to achieve a targeted n-3 PUFA intake. The adjusted PV was calculated as: (PV × 300)/(EPA + DHA), where the concentrations are the labelled content in mg/g of oil. Similarly, the adjusted anisidine value was calculated as: (AV × 300)/(EPA + DHA). Adjusted Totox was calculated as: (2 × AdjPV) + AdjAV. Associations between variables of interest were assessed using simple linear correlations and Spearman's rank correlations. Parameters measured were compared between countries of origin (New Zealand, Australia, and Other) using general linear regression models.

## Results

We initially studied 32 individual fish oil supplements available in the New Zealand market (Table 1). An extra four products were later identified and also examined, but did not have their EPA + DHA content measured (Table 1). There was a considerable variation in price among different brands, with the recommended retail price (RRP) per gram of fish oil varying from $0.05 to $0.77 (USD) (Table 1).

### EPA + DHA content

**Figure 1:**
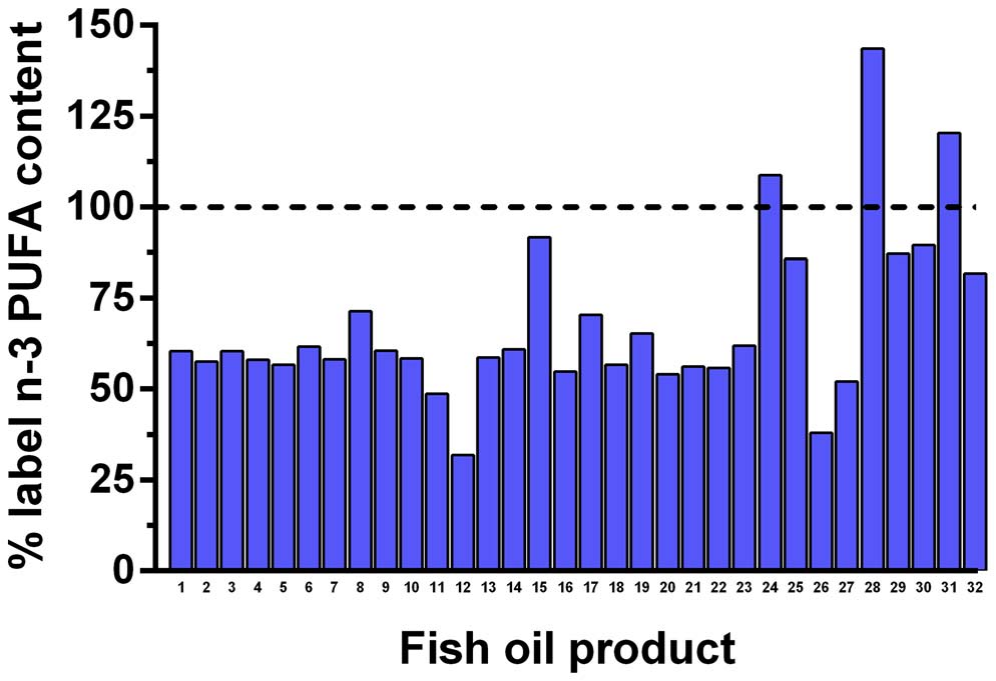
The actual n-3 PUFA content (EPA + DHA) contained in individual retail fish oil products in relation to the claimed content (dotted line).

The total oil content of individual capsules exceeded 97% of the labelled oil content for all supplements tested (Table 1). However, there was a marked disparity between the label-claimed content of EPA + DHA and the actual capsule content of these fatty acids (Table 1; Figure 1), with supplements containing on average 68% (SD = 23%) of the claimed content. Only 3 of the 32 oils tested contained a quantity of EPA + DHA that was equal to or higher than that claimed by the label, with more than two-thirds of supplements tested (22; 69%) containing less than 67% (Table 1; Figure 1). Two supplements contained approximately one third of the label concentrations of EPA + DHA (Table 1; Figure 1).

There was some indication that the more expensive brands had more accurate labelling of EPA + DHA content, as RRP was positively correlated with the ratio of measured to claimed EPA + DHA (ρ = 0.55; p = 0.001). In addition, despite the majority of fish oils having less n-3 PUFA than claimed, the label EPA + DHA content was well correlated with the actual n-3 PUFA content (ρ = 0.71; p < 0.0001).

### Oxidative markers

**Figure 2:**
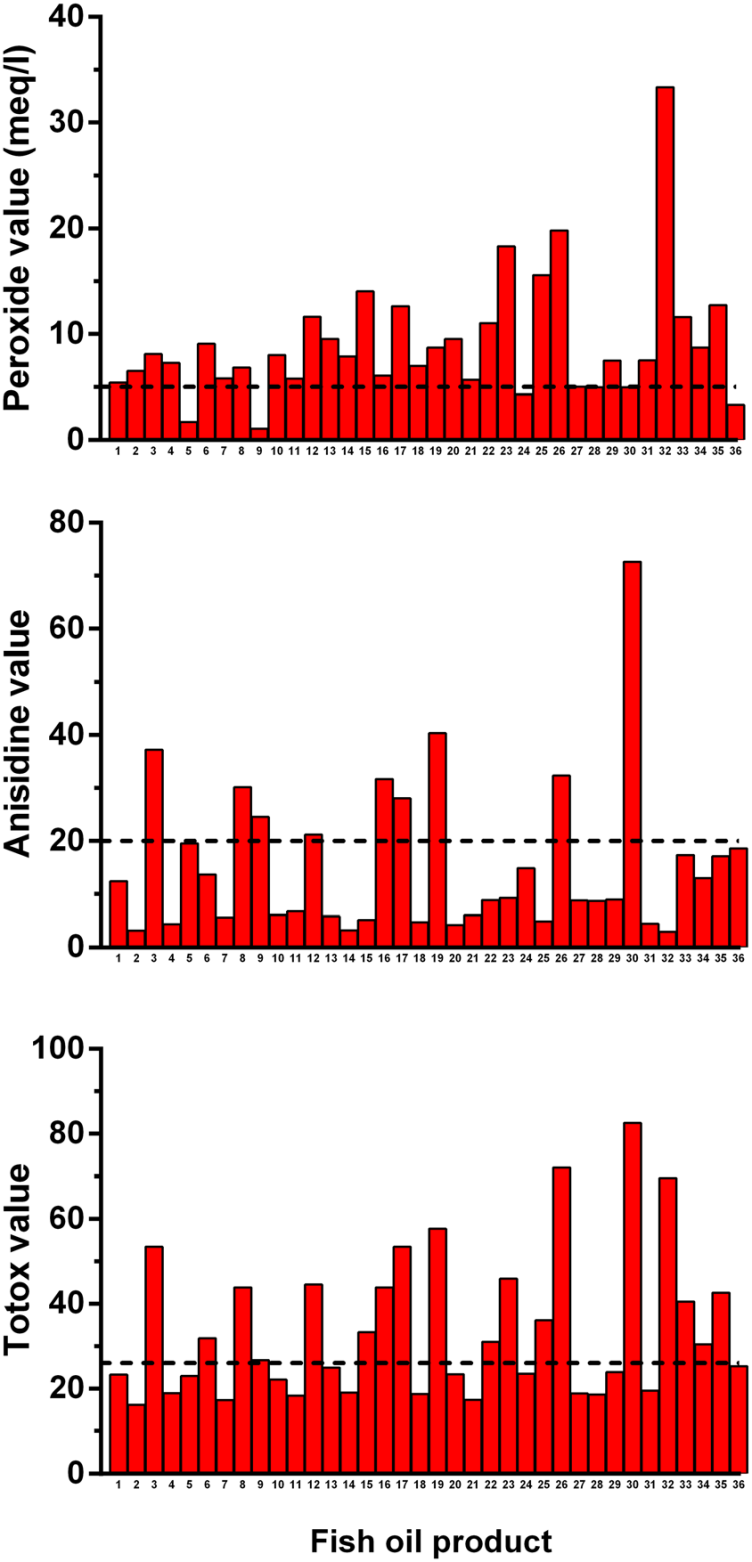
The content of oxidation markers in retail fish oil tested in relation to recommended international thresholds (dotted lines).

There were high levels of oxidation in the fish oils assessed (Table 1), with the vast majority of supplements tested failing to meet recommended levels of oxidation markers (Figure 2). 30/36 (83%) products exceeded the recommended PV, 9/36 (25%) exceeded AV, and 18/36 (50%) exceeded recommended Totox thresholds (Figure 2). Only 3 of 36 oils tested (8%) met all the international recommendations, not exceeding any of these indices (Figure 2). After adjustment for concentration, only 19% (7/36) were within the recommended limits, indicating that the high frequency of excess oxidation was not simply an artefact caused by the concentrated oils.

There were no associations between the time to best-before-date and concentrations of oxidation markers (data not shown). There was a positive correlation between RRP and both PV (ρ = 0.41; p = 0.013) and Totox (ρ = 0.39; p = 0.018). However, this association likely reflected increased n-3 concentrations in more expensive products, as there was no association between RRP and content-adjusted PV (p = 0.34) and Totox (p = 0.46).

**Figure 3:**
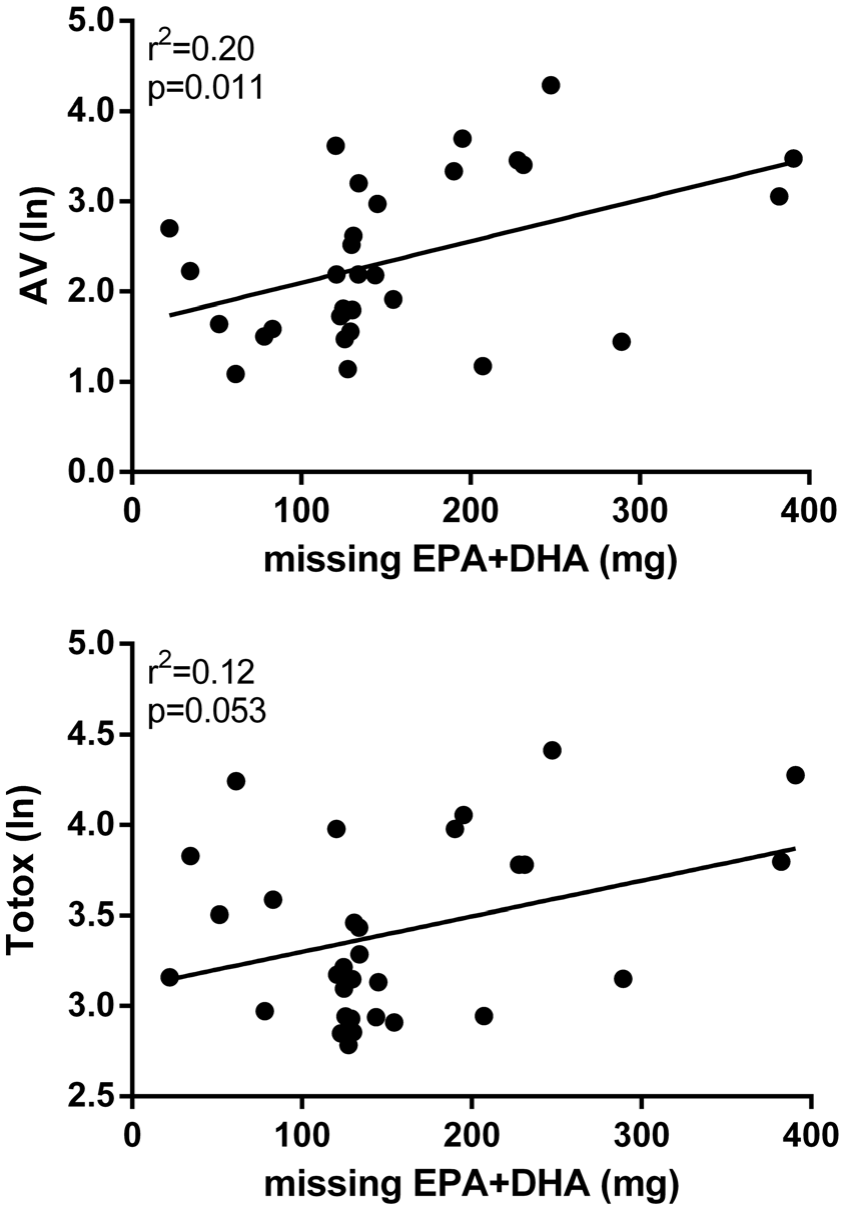
The association between missing EPA + DHA (i.e. label claimed minus actual content) and both anisidine value (AV) and Totox. AV and Totox data have been log-transformed.

There was an observed association between the missing EPA + DHA (i.e. label claimed minus actual content) and both AV (r = 0.45; p = 0.011) and Totox (r = 0.35; p = 0.053) (Figure 3). Missing EPA + DHA content was not associated with PV (r = 0.06; p = 0.75).

### Country of origin

Fish oil supplements manufactured in countries other than New Zealand or Australia were considerably more expensive according to the RRP per gram of fish oil. Supplements from other countries cost on average $0.57 in comparison to $0.13 (USD) from New Zealand (p < 0.0001) and 25 cents from Australia (p < 0.0001). However, these international supplements were not associated with better supplement quality. The percentage EPA + DHA content (in proportion to claimed label content) was virtually identical in supplements from the three sources, and there were no differences in levels of any oxidative markers assessed (data not shown).

## Discussion

This study shows that almost all fish oil supplements available in the New Zealand market contain much lower concentrations of long chain n-3 PUFAs than claimed by the product label. Importantly, the majority of fish oils exceeded the recommended indices of oxidative markers.

The discrepancy between actual and labelled n-3 PUFA content is consistent with a study of fish oils sold at retail in South Africa, and an older international survey where products were purchased in Canada, USA and UK; both found that more than half of the marine n-3 PUFA products surveyed contained less than 90% of the n-3 PUFAs claimed^18^,^19^. A small study in Ohio showed even greater discrepancy^20^. In contrast, an Australian survey of retail products showed relatively close agreement between the labelled and measured n-3 PUFA content^21^. However, the method used to convert qualitative data to capsule content in that survey did not take into account the glycerol content of the oil, and would therefore overestimate the n-3 PUFA content.

As the labelled content of most fish oil products was inaccurate, it is important to consider how and when the n-3 PUFA content had been measured. The product labels did not indicate the method used to measure n-3 PUFA content, or at what stage of production this occurred. However, it is interesting that 17 products listed the same concentration of n-3 PUFAs (180 mg EPA and 120 mg DHA per gram of oil). Similar concentrations would be expected given that most oils are sourced from oily fish from the west coast of South America. However, such striking uniformity suggests that the companies selling these products have relied on data provided by extractors who supply fish oil to multiple brands. Thus, the labelled composition may not reflect the final n-3 PUFA content of product, which may have changed due to oxidation during transport, encapsulation, packing, and storage. Further, while there is evidence of seasonal variation in the n-3 PUFA content of oily fish^22^, the labelled n-3 PUFA content of individual brands does not vary with the best before date (which is related to the date of manufacture). This suggests that the fatty acid content is unlikely to be measured in individual batches of fish oil.

The high levels of oxidation identified in this study are broadly consistent with other surveys, which have shown that 11–62% of fish oil products are oxidised above international recommendations^15^,^23^,^24^,^25^,^26^. As oil oxidises, the concentrations of EPA and DHA (the purported active compounds) decrease, suggesting reduced efficacy. In our study, oxidation may at least in part account for the low n-3 PUFA levels observed as there was an association between AV and Totox and the missing n-3 PUFA. Note that it is not surprising that PV was not associated with missing n-3 PUFA as lipid peroxides are initially formed during oxidation but subsequently broken down over time to secondary oxidation products, such that a low PV is consistent with both minimal and severe levels of oxidation. Nonetheless, the relatively weak correlations between these measures suggest that other unknown factors are also important, which could include poor quality control during manufacture.

The health implications of oxidised fish oil consumption remain unclear^14^,^27^. There is some evidence that specific n-3 PUFA oxidation products (i.e. resolvins, protectins and n-3 PUFA derived isoprostanes) have a role in mediating the anti-inflammatory effects of n-3 PUFA supplementation^28^,^29^. However, it is not clear whether these anti-inflammatory oxidation products are produced in sufficient quantities during capsule storage, to confer a net benefit to consuming oxidised fish oil. In fact, evidence from animal studies show that large doses of oxidised lipids may cause organ toxicity^30^, growth retardation^30^, and accelerated atherosclerosis^31^. Only one relatively short study has compared the effects of oxidised and unoxidised fish oil in humans^32^, observing no evidence of acute oxidative toxicity. However, the effects of long-term exposure to oxidised oils (particularly on markers associated with atherosclerosis) have not been studied. To our knowledge, the oxidative state of fish oils adopted in clinical trials has never been reported, so the possible effects of oxidation on trial outcomes are unknown^14^. It is possible that the conflicting and often disappointing results in clinical trials may have resulted from the use of highly oxidised supplements^14^. The high levels of oxidation shown in this study underline the importance of understanding the effects of oxidised fish oil taken in dietary supplements, particularly for pregnant women, children, and consumers with inflammatory, metabolic, or cardiovascular disease.

Our results also indicate that consumers would be unable to identify unoxidised fish oil supplements. The time until the best before date printed on the packaging had no relationship to the level of oxidation. Cost was also unhelpful, for while there was a correlation between cost and oxidation such that the more expensive supplements had greater oxidation, this relationship was an artefact of concentration (concentrated supplements were more expensive). In addition, supplements from outside Oceania were as oxidised as those manufactured in Australia and New Zealand. Even the two products that could only be purchased after naturopathic consultation had excess oxidation, and though one was labelled with the peroxide value, the true PV exceeded the label by more than four-fold.

It is important to emphasise that all products were well within their best-before dates, with the shortest being 270 days from analysis. The range of best-before dates observed in this study reflects the substantial variability presented on retail shelves. All retailers with a physical store were observed to keep their oil under the same conditions; at room temperature under artificial lighting, (the storage conditions of online only stores could not be observed). As fish oil products were selected based on predefined rules that were applied to all supplements tested, selection bias is unlikely. The major limitation of this study is that each product was only purchased from a single store, so that our study would have been unable to identify variations between batches or stores. Nevertheless, this study represents a comprehensive sample of the fish oil products available to consumers in New Zealand at the time of sampling. As most fish oil products sold globally (including New Zealand) appear to be sourced from deep sea fish from the west coast of South America, the results of our survey are likely to have international relevance.

In this study, we included all identified fish oil products (the most popular form of n-3 PUFA supplement), but not n-3 PUFA supplements produced from other marine sources such as krill. There are important differences in the composition of krill oils, particularly in the incorporation of n-3 PUFA as phospholipids^33^ and the presence of naturally occurring antioxidants such as the pigment astaxanthin^34^. These differences may affect the propensity of these oils to oxidise^7^. Thus, the results of our study cannot be generalised to other marine oils that are not sourced from fish, and krill oils should be studied separately.

In summary, the majority of fish oil supplements available for purchase in New Zealand not only have n-3 PUFA contents well below those claimed by labels, but are also considerably oxidised (with PV, AV, and Totox values above recommended levels). The associated health implications are unclear. Future studies should investigate the effect of environmental conditions on oxidation of encapsulated fish oils, particularly regarding the oxidation process when supplements are stored in retail or home environments. Further, clinical trials investigating the health effects of fish oil products should measure and report their peroxide and anisidine values, so that the importance of oxidation to efficacy and harms can be better understood.

## Author Contributions

B.B.A., W.S.C., P.L.H., D.C.-S. and J.G.B.D. conceived and designed the study. B.B.A. performed chemical analyses with assistance from S.T. and S.G.V.-B. and compiled the data. M.L.G. took responsibility for GC analysis. J.G.B.D. carried out the statistical analyses and compiled the results. B.B.A., J.G.B.D. and W.S.C. wrote the manuscript with input from all other authors.

## Figures and Tables

**Table 1:** List of retail fish oils tested. Adj, adjusted for labelled n-3 PUFA content to reflect the oxidation products per 300 mg combined EPA + DHA; AV, anisidine value; BBD, time to best before date as per label; PV, peroxide value; RRP, recommended retail price in US dollars per gram of fish oil (equivalent to one capsule in most cases); Totox, total oxidation value. Note: the country of origin reflects the country claimed on the product packaging or by a company representative. It reflects the site of encapsulation or packaging; the vast majority of oils were sourced from South American deep sea fish. International guidelines recommend values for PV, AV, and Totox of <5 meq/l, <20, and <26, respectively

		RRP	BBD	Oil mass per capsule		EPA + DHA per capsule		PV			Adj PV		
Code	Country of origin	(US$)	(days)	Actual (g)	% label	Label (mg)	Actual (mg)	(meq/l)	AV	Totox	(meq/l)	Adj AV	Adj Totox
1	Australia	0.14	318	1.064	106.4	300	181	5.43	12.43	23.29	5.43	12.43	23.29
2	Australia/NZL	0.11	321	1.000	100.0	300	173	6.52	3.14	16.17	6.52	3.14	16.17
3	NZL	0.14	553	1.009	100.9	300	181	8.11	37.22	53.43	8.11	37.22	53.43
4	NZL	0.10	561	1.000	100.0	300	174	7.30	4.37	18.97	7.30	4.37	18.97
5	NZL	0.15	349	1.096	109.6	300	170	1.71	19.53	22.95	1.71	19.53	22.95
6	Australia	0.13	438	1.094	109.4	300	185	9.08	13.72	31.88	9.08	13.72	31.88
7	NZL	0.06	765	0.985	98.5	300	174	5.82	5.64	17.29	5.82	5.64	17.29
8	Australia	0.40	495	1.167	97.3	694	495	6.85	30.17	43.87	2.96	13.04	18.96
9	Australia	0.11	587	1.094	109.4	300	182	1.09	24.54	26.72	1.09	24.54	26.72
10	Australia	0.12	800	1.002	100.2	300	175	8.00	6.11	22.12	8.00	6.11	22.12
11	NZL	0.07	969	1.001	100.1	300	146	5.78	6.79	18.34	5.78	6.79	18.34
12	Norway	0.50	739	1.048	104.8	550	176	11.65	21.23	44.53	6.35	11.58	24.29
13	Australia	0.10	404	1.003	100.3	300	176	9.56	5.81	24.93	9.56	5.81	24.93
14	Australia	0.11	587	1.500	100.0	450	274	7.88	3.25	19.00	5.25	2.16	12.67
15	Canada	0.50	249	1.022	102.2	500	458	14.06	5.17	33.28	8.43	3.10	19.97
16	NZL	0.10	341	1.142	114.2	450	247	6.07	31.72	43.85	4.04	21.15	29.24
17	Australia	0.44	310	1.032	103.2	600	423	12.66	28.10	53.42	6.33	14.05	26.71
18	NZL	0.05	796	0.995	99.5	300	170	6.98	4.76	18.73	6.98	4.76	18.73
19	NZL	0.07	614	1.508	100.6	450	294	8.70	40.34	57.75	5.80	26.89	38.50
20	Australia	0.10	766	1.513	100.9	450	243	9.54	4.26	23.34	6.36	2.84	15.56
21	Australia	0.11	857	0.992	99.2	300	169	5.67	6.04	17.38	5.67	6.04	17.38
22	NZL	0.11	521	1.007	100.7	300	167	11.05	8.92	31.03	11.05	8.92	31.03
23	India	0.74	460	0.486	97.2	275	170	18.33	9.28	45.93	19.99	10.12	50.11
24	Australia	0.51	368	1.136	113.6	520	566	4.32	14.91	23.54	2.49	8.60	13.58
25	Australia	0.34	156	1.037	100.1	521	447	15.59	4.91	36.09	8.98	2.83	20.78
26	Australia	0.35	187	1.347	112.2	600	228	19.82	32.36	72.01	9.91	16.18	36.00
27	Norway, bottled in NZL	0.07	826	1.000	100.0	300	156	5.02	8.87	18.90	5.02	8.87	18.90
28	NZL	0.23	732	0.996	99.6	300	431	4.97	8.69	18.64	4.97	8.69	18.64
29	USA	0.51	793	1.190	99.2	600	523	7.47	8.94	23.89	3.74	4.47	11.94
30	NZL	0.22	900	1.514	100.9	900	806	4.97	72.63	82.58	1.66	24.21	27.53
31	USA	0.42	641	1.214	101.2	191	230	7.50	4.51	19.52	11.78	7.09	30.66
32	NZL	0.54	887	0.956	95.6	425	348	33.34	2.98	69.67	23.54	2.10	49.18
33	Australia/NZL	0.28	768	1.022	98.8	600	?	11.61	17.34	40.55	5.80	8.67	20.28
34	Australia/NZL	0.24	367	1.543	99.4	900	?	8.72	13.01	30.46	2.91	4.34	10.15
35	Norway	0.77	611	1.048	104.8	550	?	12.75	17.15	42.65	6.96	9.35	23.26
36	NZL	0.10	273	1.142	114.2	300	?	3.33	18.65	25.32	3.33	18.65	25.32

## References

[b1] Barnes P. M., Powell-Griner E., McFann K. & Nahin R. L. Complementary and alternative medicine use among adults: United States, 2002. Adv Data 343, 1–19, 10.1016/j.sigm.2004.07.003 (2004). 15188733

[b2] Barnes P. M., Bloom B. & Nahin R. L. Complementary and alternative medicine use among adults and children: United States, 2007. Natl Health Stat Report 12, 1–23, 10.1037/e623942009-001 (2008). 19361005

[b3] Calder P. C. Marine omega-3 fatty acids and inflammatory processes: effects, mechanisms and clinical relevance. Biochim Biophys Acta [Epub ahead of print], 10.1016/j.bbalip.2014.08.010 (2014). 25149823

[b4] Calder P. C. Omega-3 polyunsaturated fatty acids and inflammatory processes: Nutrition or pharmacology? Br J Clin Pharmacol 75, 645–662, 10.1111/j.1365-2125.2012.04374.x (2013). 22765297 PMC3575932

[b5] Karr J. E., Alexander J. E. & Winningham R. G. Omega-3 polyunsaturated fatty acids and cognition throughout the lifespan: a review. Nutr Neurosci 14, 216–225, 10.1179/1476830511Y.0000000012 (2011). 22005286

[b6] Calder P. C. n-3 Fatty acids and cardiovascular disease: evidence explained and mechanisms explored. Clin Sci 107, 1–11, 10.1042/cs20040119 (2004). 15132735

[b7] Shahidi F. & Zhong Y. Lipid oxidation and improving the oxidative stability. Chem Soc Rev 39, 4067–4079, 10.1039/b922183m (2010). 20617249

[b8] Benzie I. F. F. Lipid peroxidation: A review of causes, consequences, measurement and dietary influences. Int J Food Sci Nutr 47, 233–261, 10.3109/09637489609012586 (1996). 8735779

[b9] Zuta P., Simpson B., Zhao X. & Leclerc L. The effect of α-tocopherol on the oxidation of mackerel oil. Food Chemistry 100, 800–807, 10.1016/j.foodchem.2005.11.003 (2007).

[b10] Global Organisation for EPA and DHA Omega-3. GOED Voluntary Monograph (v.4) (2012). Available at: http://www.goedomega3.com/index.php/our-members/quality-standards. [Date of access: 04/12/2014].

[b11] US Council for Responsible Nutrition. Voluntary Monograph: Omega-3 DHA, Omega-3 EPA, Omega-3 DHA & EPA (2006). Available at: http://www.crnusa.org/pdfs/O3FINALMONOGRAPHdoc.pdf. [Date of access: 04/12/2014].

[b12] Health Canada. Monograph: Fish Oil (2009). Available at: http://webprod.hc-sc.gc.ca/nhpid-bdipsn/monoReq.do?id=88&lang=eng. [Date of access: 04/12/2014].

[b13] EFSA Panel on Biological Hazards (BIOHAZ). Scientific opinion on fish oil for human consumption. Food hygiene, including rancidity. EFSA J 8, 1874, 10.2903/j.efsa.2010.1874 (2010).

[b14] Albert B. B., Cameron-Smith D., Hofman P. L. & Cutfield W. S. Oxidation of marine omega-3 supplements and human health. BioMed Res Int 2013, 464921, 10.1155/2013/464921 (2013). 23738326 PMC3657456

[b15] Anonymous. Something fishy? Omega-3 supplements test. Consumer 469, 12–15 (2007).

[b16] European Pharmacopoeia 5^th^ edn, Vol. 2 (European Directorate for the Quality of Medicines & Healthcare, Council of Europe, 2004).

[b17] Lepage G. & Roy C. Direct transesterification of all classes of lipids in a one-step reaction. J Lipid Res 27, 114–120 (1986). 3958609

[b18] Opperman M., Marais de W. & Spinnler Benade A. J. Analysis of omega-3 fatty acid content of South African fish oil supplements. Cardiovasc J Afr 22, 324–329, 10.5830/cvja-2010-080 (2011). 22159321 PMC3721812

[b19] Ackman R. G., Ratnayake W. M. N. & Macpherson E. J. EPA and DHA contents of encapsulated fish oil products. J Am Oil Chem Soc 66, 1162–1164, 10.1007/BF02670104 (1989).

[b20] Press R. The omega-3 fatty acid composition and cost analysis of fish oil supplements: fishing for the best deals, Ohio State University, Department of Human Nutrition, Honors thesis. (2011).

[b21] Hamilton K., Brooks P., Holmes M., Cunningham J. & Russell F. D. Evaluation of the composition of omega-3 fatty acids in dietary oil supplements. Nutr Diet 67, 182–189, 10.1111/j.1747-0080.2010.01453.x (2010).

[b22] Gamez-Meza N. *et al.* Seasonal variation in the fatty acid composition and quality of sardine oil from *Sardinops sagax caeruleus* of the Gulf of California. Lipids 34, 639–642, 10.1007/s11745-999-0409-1 (1999). 10405979

[b23] Halvorsen B. L. & Blomhoff R. Determination of lipid oxidation products in vegetable oils and marine omega-3 supplements. Food Nutr Res 55, 5792, 10.3402/fnr.v55i0.5792 (2011). PMC3118035 21691461

[b24] Kolanowski W. Omega-3 LC PUFA contents and oxidative stability of encapsulated fish oil dietary supplements. Int J Food Prop 13, 498–511, 10.1080/10942910802652222 (2010).

[b25] Fierens C. & Corthout J. [Omega-3 fatty acid preparations - a comparative study]. J Pharm Belg 62, 115–119 (2007). 18269138

[b26] Fantoni C., Cuccio A. & Barrera-Arellano D. Brazilian encapsulated fish oils: Oxidative stability and fatty acid composition. J Am Oil Chem Soc 73, 251–253 (1996).

[b27] Turner R., McLean C. H. & Silvers K. M. Are the health benefits of fish oils limited by products of oxidation? Nutr Res Rev 19, 53–62, 10.1079/nrr2006117 (2006). 19079875

[b28] Serhan C. N. Pro-resolving lipid mediators are leads for resolution physiology. Nature 510, 92–101, 10.1038/nature13479 (2014). 24899309 PMC4263681

[b29] Brooks J. D. *et al.* The fatty acid oxidation product 15-A3t-Isoprostane is a potent inhibitor of NFκB transcription and macrophage transformation. J Neurochem 119, 604–616, 10.1111/j.1471-4159.2011.07422.x (2011). 21838782 PMC3640263

[b30] Esterbauer H. Cytotoxicity and genotoxicity of lipid-oxidation products. Am J Clin Nutr 57, 779S–758S (1993). 8475896 10.1093/ajcn/57.5.779S

[b31] Thiery J. & Seidel D. Fish oil feeding results in an enhancement of cholesterol-induced atherosclerosis in rabbits. Atherosclerosis 63, 53–56, 10.1016/0021-9150(87)90081-5 (1987). 3827970

[b32] Ottestad I. *et al.* Oxidised fish oil does not influence established markers of oxidative stress in healthy human subjects: a randomised controlled trial. Br J Nutr 108, 315–326, 10.1017/S0007114511005484 (2011). 22136711

[b33] Ali-Nehari A. & Chun B. S. Characterization of purified phospholipids from krill (*Euphausia superba*) residues deoiled by supercritical carbon dioxide. Korean J Chem Eng 29, 918–924, 10.1007/s11814-011-0273-4 (2012).

[b34] Grynbaum M. D. *et al.* Unambiguous detection of astaxanthin and astaxanthin fatty acid esters in krill (*Euphausia superba* Dana). J Sep Sci 28, 1685–1693, 10.1002/jssc.200500152 (2005). 16224962

