# Corrigendum: Fish oil supplements in New Zealand are highly oxidised and do not meet label content of n-3 PUFA

**DOI:** 10.1038/srep35092

**Published:** 2016-11-07

**Authors:** Benjamin B. Albert, José G. B. Derraik, David Cameron-Smith, Paul L. Hofman, Sergey Tumanov, Silas G. Villas-Boas, Manohar L. Garg, Wayne S. Cutfield

Scientific Reports 5: Article number: 792810.1038/srep07928; published online: 01 21 2015; updated: 11 07 2016

This Article contains typographical errors.

In the ‘PV, AV, and Totox’ section of the Methods, the formula for the p-anisidine value reads “10 × [1.2 × (A_2_ − A_1_)]/m” but should read “[10 x (1.2 x A_2_ - A_1_)]/m”.

The calculations used to derive peroxide value (PV) produced a concentration in meq/kg and not in meq/l as stated in the Article. “meq/l” should read “meq/kg” in the first and third paragraphs of the ‘PV, AV, and Totox’ section in the Methods; and in the title of Table 3, and the headings of columns 9 and 10 in Table 3.

